# Network efficiency in autism spectrum disorder and its relation to brain overgrowth

**DOI:** 10.3389/fnhum.2013.00845

**Published:** 2013-12-10

**Authors:** John D. Lewis, Rebecca J. Theilmann, Jeanne Townsend, Alan C. Evans

**Affiliations:** ^1^McConnell Brain Imaging Center, Montreal Neurological Institute, McGill UniversityMontreal, QC, Canada; ^2^Department of Radiology, University of CaliforniaSan Diego, La Jolla, CA, USA; ^3^Department of Neuroscience, University of CaliforniaSan Diego, La Jolla, CA, USA; ^4^Research on Aging and Development Laboratory, University of CaliforniaSan Diego, La Jolla, CA, USA

**Keywords:** autism, brain size, network analysis, connectivity, tractography, optimal wiring, scaling

## Abstract

A substantial body of evidence links differences in brain size to differences in brain organization. We have hypothesized that the developmental aspect of this relation plays a role in autism spectrum disorder (ASD), a neurodevelopmental disorder which involves abnormalities in brain growth. Children with ASD have abnormally large brains by the second year of life, and for several years thereafter their brain size can be multiple standard deviations above the norm. The greater conduction delays and cellular costs presumably associated with the longer long-distance connections in these larger brains is thought to influence developmental processes, giving rise to an altered brain organization with less communication between spatially distant regions. This has been supported by computational models and by findings linking greater intra-cranial volume, an index of maximum brain-size during development, to reduced inter-hemispheric connectivity in individuals with ASD. In this paper, we further assess this hypothesis via a whole-brain analysis of network efficiency. We utilize diffusion tractography to estimate the strength and length of the connections between all pairs of cortical regions. We compute the efficiency of communication between each network node and all others, and within local neighborhoods; we then assess the relation of these measures to intra-cranial volume, and the differences in these measures between adults with autism and typical controls. Intra-cranial volume is shown to be inversely related to efficiency for wide-spread regions of cortex. Moreover, the spatial patterns of reductions in efficiency in autism bear a striking resemblance to the regional relationships between efficiency and intra-cranial volume, particularly for local efficiency. The results thus provide further support for the hypothesized link between brain overgrowth in children with autism and the efficiency of the organization of the brain in adults with autism.

## Introduction

Brains differ dramatically in both size and structure across species. These two dimensions of variation are not independent, but large brains are not big small brains. The organization of both gray- and white-matter varies with brain size, but not in a uniform manner. Larger brain size is associated with a greater white-matter to gray-matter ratio (Rilling and Insel, 1999b; Zhang and Sejnowski, 2000), but a reduced degree of long-distance connectivity (Ringo, 1991; Rilling and Insel, 1999a; Karbowski, 2003; Changizi, 2007), as well as with increased modular structure (Changizi and Shimojo, 2005), greater surface convolutedness (Jerison, 1982; Prothero and Sundsten, 1984; Hofman, 1985), and various other morphological and cellular aspects of neural organization. Scaling laws capture much of the variation in structure in terms of brain size (Jerison, 1982; Ringo, 1991; Karbowski, 2003; Changizi and Shimojo, 2005; Changizi, 2007). However, significant structural variability remains unaccounted for by these scaling laws.

The underpinnings of these scaling relationships are not well understood, but are thought to be related to a design principle originally postulated by Ramón y Cajal: that neural circuit design is under pressure to minimize cellular costs and conduction delays (Ramón y Cajal, 1995). Increased brain size provides increased computational power, but at hugely increased cost. Neural material is expensive to construct and to operate. The human brain makes up only about 2 percent of the total body weight, but its operation is responsible for approximately 15 percent of cardiac output, 20 percent of oxygen usage, and 25 percent of glucose usage (Magistretti, 1999). These metabolic costs are largely due to the cost of neural signaling, and maintaining the resting potentials needed for neural signaling. These costs increase with membrane surface area, which increases with the number and size of the axons. Larger brains have a larger number of axons, and the longest of these axons are both longer and slightly larger in diameter than are those of smaller brains; thus the total membrane surface area is increased. Axon diameter does not increase sufficiently with brain size, however, to compensate for the increased fiber lengths, so larger brains also have longer conduction delays (Olivares et al., 2001). These greater costs and conduction delays appear to be related to at least some of the aspects of organization that scale with brain size, e.g. the reduced degree of long-distance connectivity (Ringo, 1991; Rilling and Insel, 1999a; Karbowski, 2003; Changizi, 2007).

The focus on cross-species differences, where differences in brain size can be more than 1000-fold within classes, e.g., Mammalia, and 100-fold within orders, e.g., Primates, allows relationships between brain size and structure to be apparent despite differences in structure unrelated to brain size. But, it ignores potentially important differences in developmental brain growth trajectories. There are substantial inter-species differences in rate of brain growth, and developmental trajectories can even vary considerably between individuals, e.g., brain size may differ by as much as 50% in children of the same age (Giedd, 2008). Brain size differences between adults account for some of the differences in structure (Jäncke et al., 1997; Honey et al., 2009; Lewis et al., 2009); differences in brain growth trajectories likely account for additional structural variability.

Substantial neural reorganization occurs over development. Neural development is largely a combination of over-exuberance and competition-based elimination. Large numbers of transient projections are produced during cortical development (Rakic et al., 1986; LaMantia and Rakic, 1990), and which connections are retained is determined by their metabolic demands and their ability to compete for neurotrophins (Van Ooyen and Willshaw, 1999). Due to the lesser degree of myelination in the developing brain than in the mature brain, the differences in conduction delays and metabolic costs associated with differences in fiber length will be substantially greater (Chugani et al., 1987; Paus et al., 1999; Thatcher et al., 2008). Thus, to the extent that differences in brain size during development coincide with differences in brain size in mature individuals, normal developmental processes may underlie at least some portion of the scaling relationships seen across and within species; moreover, differences in brain size during development which do not coincide with differences in brain size in mature individuals may account for a portion of the structural variability that is not accounted for by scaling laws.

This conjecture is clearly relevant to developmental disorders showing abnormalities in brain growth trajectories. Autism spectrum disorder (ASD) is such a case. ASD is a disorder of neural developmental defined by impairments in reciprocal social interactions, impairments in verbal and non-verbal communication, and a restricted repertoire of activities and interests (American Psychiatric Association, 1994). The aetiology of ASD is unknown, but there is now consensus that brain size during development is increased. Infants who go on to a diagnosis of ASD show abnormally rapid brain growth during the first years of life (Lainhart et al., 1997; Redcay and Courchesne, 2005), and after the second or third year of life children with ASD show increased head size (Lainhart et al., 1997; Hazlett et al., 2005) and brain size (Piven et al., 1995; Courchesne et al., 2001; Hazlett et al., 2005). Early in development this size difference can be multiple standard deviations above the norm (Redcay and Courchesne, 2005).

Lewis and Elman (2008) have shown via computational modeling that the increased conduction delays presumably associated with the early brain overgrowth in ASD may lead to the later functional and structural long-range underconnectivity. Further, in adults with ASD, Lewis et al. (2012) have shown that callosal tract length adjusted for intra-cranial volume (ICV), an index of maximum brain-size during development (Whitwell et al., 2001; Aylward et al., 2002; Buckner et al., 2004), shows the typical inverse relation to relative corpus callosum size, and so the early brain overgrowth in autism appears to in fact account for some portion of the later observed long-range underconnectivity.

In the current paper we extended this work to assess the impact of the early brain overgrowth in ASD on overall brain organization. We performed a network analysis and assessed the relation between the network metrics and ICV. Network analysis methods have evolved over the past decade and a half, from straightforward applications of graph theory, which assess only network topology (Watts and Strogatz, 1998), to more sophisticated approaches which take account of the spatial aspects of connectivity to assess the efficiency of information transfer within the network (Latora and Marchiori, 2001, 2003; Achard and Bullmore, 2007; Bullmore and Sporns, 2012). Such approaches utilize measures of the length and strength of connections between all pairs of anatomical regions to estimate how efficiently information can be transferred between regions. We used probabilistic tractography to estimate the strength of connectivity between all pairs of regions, and the length of the connections between regions. We computed the efficiency of communication from all regions to all others, and within local neighborhoods. We then assessed the relation between both of these measures of efficiency and ICV, as well as group differences in efficiency. We predicted that there would be an inverse relation between ICV and both measures of efficiency, reflecting an adverse effect of brain overgrowth on overall brain organization, and that this would explain a portion of the group differences in efficiency.

## Methods

### Participants

A total of 44 adult males participated in the study: 22 with ASD ranging between 19 and 51 years of age (mean 34.14; *SD* 10.67), and 22 typical adult males ranging between 20 and 45 years of age (mean 32.25; *SD* 9.98). All ASD participants met diagnostic criteria for ASD on the DSM-IV as confirmed by a licensed clinician. Eighteen of the twenty two ASD participants met the DSM diagnosis for autistic disorder (classic autism) and, based on absence of early language delay and no significant abnormality in communication, four of the twenty two subjects additionally met diagnostic criteria for Asperger's disorder. Autism Diagnostic Interview, Revised (ADI-R) scores were available for 16 of the ASD participants; Autism Diagnostic Observation Schedule (ADOS) scores were available for 18; and Childhood Autism Rating Scale (CARS) scores were available for 12. Table 1 summarizes these data. In all but one case the ASD diagnosis was confirmed by all of the available additional assessments; the one exception was below the cutoff for the CARS, but above all cutoffs for the ADI-R and ADOS. General intellectual ability in the ASD participants was evaluated by the Wechsler Adult Intelligence Scale-Revised (WAIS-R) or the Wechsler Abbreviated Scale of Intelligence (WASI). Mean scores were: Verbal IQ, 88.48 ± 23.06; Performance IQ, 106.10 ± 15.91. Individuals with a history of significant medical or neurological disorders including seizures or with Fragile X syndrome were excluded from the sample. Typical participants with a first degree relative with a diagnosis of ASD were excluded from the sample. The participants were those from Lewis et al. (2012) augmented by new data from individuals with ASD. Those subjects who were capable gave informed consent; a caregiver gave informed consent for the others. The study was approved by the Human Research Protections Program at the University of California, San Diego.

**Table 1:** **The behavioral data**.

	**Cutoff**	**Range**	**Mean**	***SD***
ADI-R Social	10	13–54	27.06	8.74
ADI-R Communication (Verbal)	8	6–25	18.75	4.43
ADI-R Repetitive behaviors	3	3–14	8.25	2.91
ADOS Social	6	4–20	10.50	3.60
ADOS Communication	3	2–9	6.39	1.85
ADOS Stereotyped behavior		0–13	2.29	2.97
CARS	30	23.5–51.5	36.46	7.46

Cutoff scores for the Autism Diagnostic Interview, Revised (ADI-R) and the Childhood Autism Rating Scale (CARS) are available only for autism; thus we also used the autism cutoffs for the Autism Diagnostic Observation Schedule (ADOS).

### Imaging

All subjects were scanned at the UCSD Center for fMRI on a GE Signa EXCITE 3.0T short bore scanner with an eight-channel array head coil. Three types of images were acquired from each subject: (i) one set of 3D *T*_1_-weighted images (Fast Gradient Echo, SPGR;*TE* = 3.1 ms; flip angle = 12; NEX = 1; FOV = 25 cm; matrix = 256 × 256); (ii) two sets of diffusion weighted images isotropically distributed along 15 directions (dual spin-echo,EPI; *TR* = 15 s; TE = 89 ms; 45 axial slices; NEX = 2; FOV = 22 cm; matrix = 128 × 128; resolution = 1.875 × 1.875 × 3 mm; 3 mm interleaved contiguous slices; *b* value = 1400 s/mm^2^); and (iii) fieldmaps matched to the diffusion-weighted images. During acquisition scans were visually inspected to ensure that usable data were collected. Where motion introduced visible artifacts in multiple volumes, the scan sequence was aborted and reinitiated, or an additional scan was acquired. Note that at least two sets of diffusion weighted images were acquired, each with NEX = 2; thus each image was acquired at least four times. Fieldmaps were acquired before the first diffusion-weighted images were acquired, and, in cases where there was between scan motion, an additional set of fieldmaps was acquired after the second.

### Image processing

**Figure 1:**
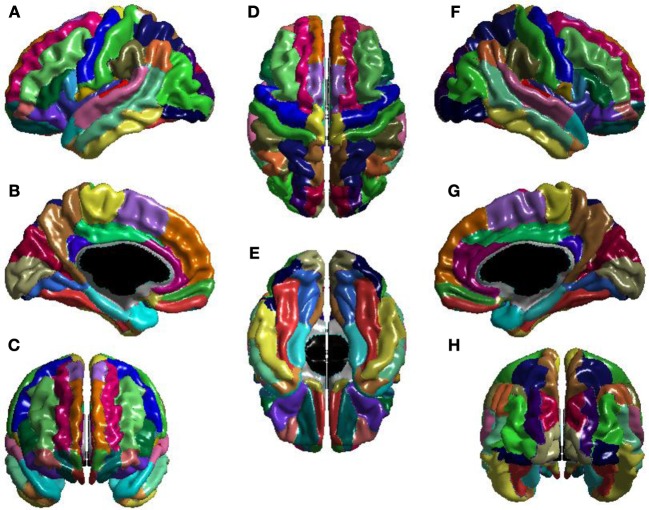
**The AAL atlas views: (A) left lateral, (B) left medial, (C) anterior, (D) superior, (E) inferior, (F) right lateral, (G) right medial, and (H) posterior.** The regions are colored to identify region boundaries. Similarity of colors between spatially separated regions is not meaningful; all regions are spatially contiguous. The cortical parcellation is based on anatomical landmarks, e.g., sulci. There are 78 cortical regions. These cortical regions serve as the nodes of the network.

The *T*_1_-volumes were processed with CIVET, a fully automated structural image analysis pipeline developed at the Montreal Neurological Institute. CIVET corrects intensity non-uniformities using N3 (Sled et al., 1998); aligns the input volumes to the Talairach-like ICBM-152-nl template (Collins et al., 1994); classifies the image into white matter, gray matter, cerebrospinal fluid, and background (Zijdenbos et al., 2002; Tohka et al., 2004); and extracts the white-matter and pial surfaces (Kim et al., 2005). ICV was calculated via the atlas based spatial normalization procedure described in Buckner et al. (2004). The CIVET results were visually inspected to ensure that surface construction was correct, and then used to construct the seed, stop, and target masks for use with FSL's *probtrackx* (Behrens et al., 2007). Seed masks control from which voxels tracts are seeded; seed masks were white-matter. Stop masks determine where tract propagation is halted; stop masks were voxels on the boundary of white-matter. Target masks determine the mapping from voxels of the stop masks to brain regions; target masks were the voxels at the boundary of white-matter and the cortex, and mapped these voxels to the Automatic Anatomical Labeling (AAL) atlas (Tzourio-Mazoyer et al., 2002), shown in Figure 1.

Each diffusion dataset was first corrected of distortions caused by inhomogeneities in the magnetic field using the fieldmaps. This was done using software developed by the UCSD Center for fMRI. The resulting diffusion-weighted volumes were then subjected to a quantitative quality control evaluation using *DTIprep* (Liu et al., 2010). *DTIprep* corrects motion artifacts where possible, and excludes directions from the data when correction is not possible. For each subject, the two diffusion-weighted volumes with the fewest number of excluded directions were chosen for further processing. The *b*0 volumes of both diffusion scans were then affine registered to the *T*_1_-volume in stereotaxic space using the Oxford University FMRIB Software Library's (FSL) *flirt* (Jenkinson and Smith, 2001), and the resultant transforms used to align the two 4D volumes; the rotational component was applied to the directional vectors. The two were then merged using FSL's *fslmerge*. The merged volume was then preprocessed for probablistic tractography with FSL's *bedpostx* (Behrens et al., 2007). Probabilistic tractography, utilizing FSL's *probtrackx* with distance-bias correction (Behrens et al., 2003, 2007), was then seeded from 10,000 random locations within each voxel of the seed masks to generate the number of tracts connecting voxels in the target mask. A native-scale 4D diffusion volume was generated using the same procedure, but with the scaling component removed from the transforms; this was processed in the same way to generate the lengths of the connections between voxels in the target mask. These results were then compiled for each AAL region generating matrices of the total number of connections between each pair of AAL regions, and the mean length of those connections. The total number of connections between each pair of AAL regions was then divided by the mean size of the two AAL regions to provide an index of the strength of connectivity between pairs of regions.

### Analysis

The efficiency of communication was calculated for all regions, based on the definition provided by Latora and Marchiori (2001, 2003). The relation of ICV to efficiency was assessed with statistical linear models, as well as group differences in efficiency. Correction for multiple comparisons was done using the false discovery rate method (Benjamini and Hochberg, 1995).

Latora and Marchiori (2001) defined the efficiency ε_*ij*_ in the communication between nodes *i* and *j* to be inversely proportional to the shortest path length *d*_*ij*_ between nodes *i* and *j*. They take the shortest path length *d*_*ij*_ to be the smallest sum of the physical distances throughout all of the possible paths from *i* to *j* in the graph, i.e., the travel distance, not the number of edges nor the Euclidean distance. The efficiency of a network, *G*, is then

$$E(G)=\frac{\sum_{i\;\neq \;j\;\in \;G}\varepsilon_{ij}}{N(N-1)}=\frac{1}{N(N-1)}\sum_{i\;\neq \;j\in \;G}\frac{1}{d_{ij}}$$

where *N* is the number of nodes in the network graph *G*; ε_*ij*_ is the efficiency of the connection between nodes *i* and *j*; and *d*_*ij*_ is the length of the shortest path, in terms of physical distances, between nodes *i* and *j*. This measure is normalized by *E*(*G*_*IDEAL*_), the fully connected network. Note that the measures of efficiency take into account the physical distances involved in information transfer, and so relate more closely to the neurobiological substrates than do purely topological measures (Watts and Strogatz, 1998; Achard and Bullmore, 2007; Rubinov and Sporns, 2010).

Latora and Marchiori (2001) apply this formulation to both the entire network, which they refer to as *global efficiency*, and to the subnetworks of the immediate neighbors of each node; they define *local efficiency* as the mean of *E*(*G*_*i*_), for all nodes *i*, where *G*_*i*_ is the subgraph of all the neighbors of node *i*. These definitions give a single measure of *local efficiency* and of *global efficiency* for the entire network. But, the definitions can be given straightforward translations to provide measures of efficiency for each node, or for collections of nodes. Achard and Bullmore (2007) define *nodal efficiency*, which we will refer to as *nodal global efficiency*, as the inverse of the harmonic mean of the minimum number of edges between a node, *i*, and all other nodes in the network. Utilizing the physical distances, as per Latora and Marchiori (2001), the *nodal global efficiency* of node *i* is thus

$$E_{nodal\;global}(G,i)=\frac{\sum_{j\;\in \;G,i\;\neq \;j}\varepsilon_{ij}}{\left(N-1\right)}=\frac{1}{\left(N-1\right)}\sum_{j\;\in \;G,\;i\;\neq \;j}\frac{1}{d_{ij}}$$

where *N* is the number of nodes in the network graph *G*; ε_*ij*_ is the efficiency of the connection between nodes *i* and *j*; and *d*_*ij*_ is the length of the shortest path, in terms of physical distances, between nodes *i* and *j*. The definition of *local efficiency* can likewise be parsed to provide a measure of *nodal local efficiency*; recall that the *local efficiency* of a network is the mean of *E*(*G*_*i*_), for all nodes *i*, where *G*_*i*_ is the subgraph of all the neighbors of node *i*. Thus, the *nodal local efficiency* of node *i* is simply

$$E_{nodal\;local}(G,i)=\frac{\sum_{j\;\neq \;k\;\in \;G_{i}}\varepsilon_{jk}}{N_{G_{i}}(N_{G_{i}}-1)}=\frac{1}{N_{G_{i}}(N_{G_{i}}-1)}\sum_{j\;\neq \;k\;\in \;G_{i}}\frac{1}{d_{jk}}$$

where *N*_*Gi*_ is the number of nodes in the subgraph *G*_*i*_ consisting of all of the neighbors of *i;* ε_*jk*_ is the efficiency of the connection between nodes *j* and *k*; and *d*_*jk*_ is the length of the shortest path, in terms of physical distances, between nodes *j* and *k*.

These definitions treat connections in a binarized fashion, i.e., as either existing or not. But, the strengths of the connections reflect, albeit poorly, biophysical properties of the underlying axons that are related to conduction velocity and metabolic costs, e.g., myelination. Moreover, weak long-range connections between strongly connected modules have been argued to provide the shortcuts that make the brain an efficient small-world architecture (Gallos et al., 2012). The strengths of the connections in the brain may thus be critical to an accurate assessment of its efficiency. Therefore, we utilize a version of these measures that incorporates connection weight, i.e., the total number of tracts connecting two regions, corrected for the distance-bias and region size. Based on Rubinov and Sporns (2010), we define the *weighted distance* between nodes *i* and *j* as

$$d_{ij}^{w}=\sum_{\forall e\in S}\frac{l_{e}}{w_{e}}$$

where *S* is the set of edges in the shortest path between nodes *i* and *j; l*_*e*_ is the length of edge *e*; and *w*_*e*_ is the connection weight for edge *e*. Also based on Rubinov and Sporns (2010), our weighted formulations of *nodal global efficiency* and *nodal local efficiency* are

$$E_{nodal\;global}^{weighted}(G,i)=\frac{1}{\left(N-1\right)}\sum_{j\;\in \;G,\;i\;\neq \;j}{\left(d_{ij}^{w}\right)}^{-1}$$

where *N* is the number of nodes in the network graph *G;* and *d*^*w*^_*ij*_ is the shortest path, in terms of *weighted distance*, between nodes *i* and *j*; and

$$E_{nodal\;local}^{weighted}(G,i)=\frac{1}{N_{G_{i}}(N_{G_{i}}-1)}\sum_{j\;\neq \;k\;\in \;G_{i}}{\left({\left(d_{jk}^{w}\right)}^{-1}w_{ij}w_{ik}\right)}^{1/3}$$

where *N*_*Gi*_ is the number of nodes in the subgraph *G*_*i*_ consisting of all of the neighbors of *i;* is the shortest path, in terms of *weighted distance*, between nodes *j* and *k*; and *w*_*ij*_ and *w*_*ik*_ are the connection weights between nodes *i* and *j*, and *i* and *k*, respectively. As per Latora and Marchiori (2001), these measures are normalized by considering the fully connected network.

The impact of maximum brain size during development on efficiency was assessed, as well as the group differences in efficiency. As per Lewis et al. (2012), we used ICV as an index of maximum brain size during development (Whitwell et al., 2001; Aylward et al., 2002; Buckner et al., 2004). The relation between ICV and efficiency was assessed via statistical linear models, controlling for age and total brain volume. Group differences in efficiency were assessed via statistical linear models, controlling for age. Potential group differences in the relationships between ICV and measures of efficiency were assessed by considering the group x ICV interaction term in models with both terms. In all cases, correction for multiple comparisons was done using the false discovery rate method (Benjamini and Hochberg, 1995).

## Results

**Figure 2:**
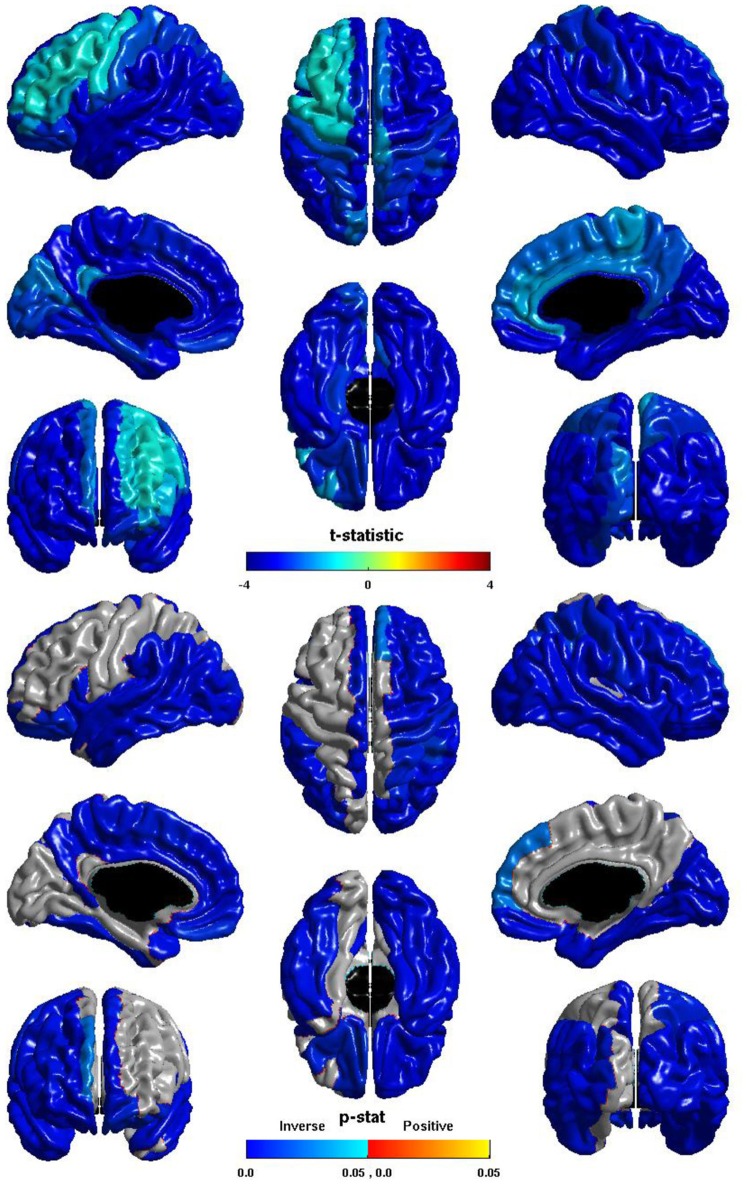
**Nodal Local Efficiency and ICV.** The *t*-statistic **(top)** and the *p*-statistic **(bottom)** for the relation between ICV and *nodal local efficiency* in each region of the AAL atlas. A negative *t*-statistic represents decreasing *nodal local efficiency* with increasing ICV. The *t*-statistic is overwhelmingly negative. The *p*-statistic is FDR-corrected, and is blue where the inverse relation is significant, and orange where a positive relation is significant. No regions show a significant positive relation. Significant inverse relations are seen bilaterally over the temporal lobes, the angular and supramarginal gyri, the pars opercularis, orbital frontal cortex, and the superior frontal gyrus; the right hemisphere shows this inverse relation more extensively over the frontal and parietal lobe; the left hemisphere shows the relation more extensively on the medial surface.

The relation between ICV and *nodal local efficiency* is shown in Figure 2. The *t*-statistic is negative over the entire cortex, thus for all regions this is an inverse relation: larger ICV is associated with less *nodal local efficiency*. This inverse relation is significant over almost the entirety of the posterior of the brain, and also the right hemisphere frontal lobe. The relation is conspicuously less negative over left dorsal lateral frontal cortex, and does not reach significance over much of left hemisphere dorsal lateral cortex; the inverse relation is stronger over the medial surface, and is significant over much of the medial surface of either hemisphere.

The ICV ^*^ group interaction term was non-significant in all regions, thus this inverse relation between ICV and *nodal local efficiency* does not differ between individuals with ASD and typical controls.

**Figure 3:**
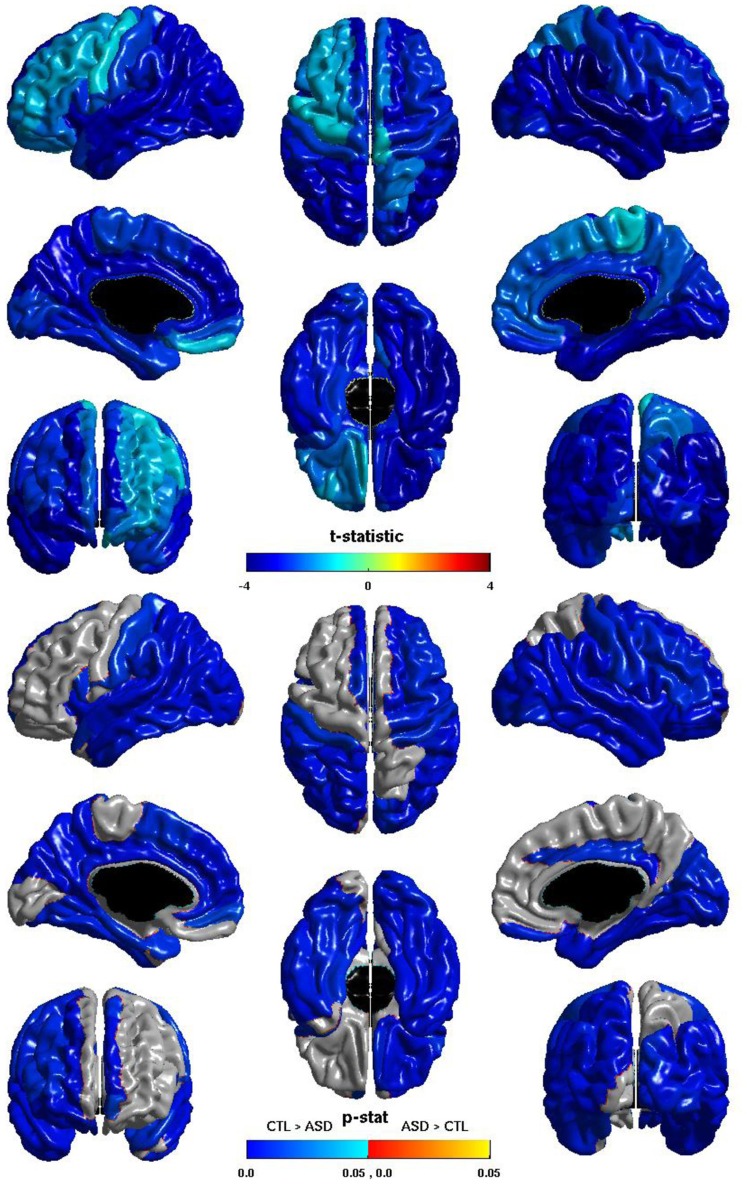
**Nodal Local Efficiency and Group.** The *t*-statistic **(top)** and the *p*-statistic **(bottom)** for the group difference in *nodal local efficiency* in each region of the AAL atlas. A negative *t*-statistic represents reduced efficiency in ASD. The *t*-statistic is negative everywhere. The *p*-statistic is FDR-corrected, and is blue where there is a significant reduction in *nodal local efficiency* in ASD, and orange where there is a significant increase in ASD. No regions show significantly increased nodal local efficiency in ASD. Significant reductions are seen bilaterally in the temporal, occipital, and parietal lobes, and in the pars opercularis; the right hemisphere additionally shows reductions over lateral regions of the frontal lobe; the left hemisphere shows more extensive reductions over the medial surface. Note the similarities to the relation of *nodal local efficiency* and ICV.

The group differences in *nodal local efficiency* are shown in Figure 3. The *t*-statistic is negative over the entire cortex, thus for all regions *nodal local efficiency* is reduced in individuals with ASD. This reduction is significant over almost the entirety of the posterior of the brain, and also the right hemisphere frontal lobe. The *t*-statistic is conspicuously less negative over left lateral frontal cortex, and the group difference does not reach significance over much of the left lateral frontal cortex; the difference is significant over much of the left medial surface. The group difference is non-significant for most of the right medial surface anterior to the cuneus. Note that the pattern of group differences in *nodal local efficiency* parallels that of the inverse relation between ICV and *nodal local efficiency*. The cosine similarity of the two *t*-statistic vectors is 0.9848.

**Figure 4:**
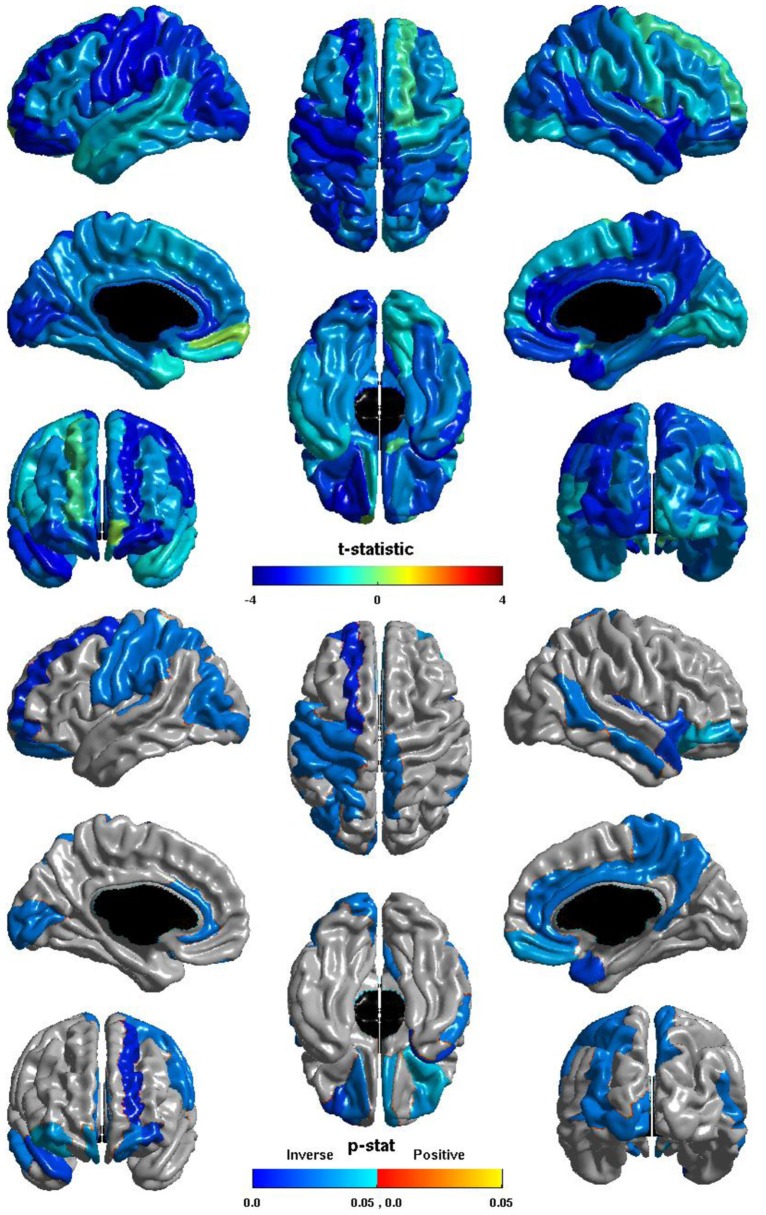
**Nodal Global Efficiency and ICV.** The *t*-statistic **(top)** and the *p*-statistic **(bottom)** for the relation between ICV and *nodal global efficiency* in each region of the AAL atlas. A negative *t*-statistic represents decreasing *nodal global efficiency* with increasing ICV. The *t*-statistic is predominately negative. The *p*-statistic is FDR-corrected, and is blue where the inverse relation is significant and orange where a positive relation is significant. No regions show a significant positive relation. Significant inverse relations are seen in the left occipital, parietal, and frontal lobes, and in primary auditory cortex; in the right temporal lobe, precuneus, and paracentral lobule; and bilaterally in the cingulate and orbitofrontal cortex.

The relation between ICV and *nodal global efficiency* is shown in Figure 4. The *t*-statistic is negative over most of the cortex, thus this is again generally an inverse relation: larger ICV is associated with less *nodal global efficiency*. Significant inverse relations are seen in the left hemisphere in all lobes, notably in visual cortex, the pre- and post-central gyri, and in primary auditory cortex; significant inverse relations are seen in the right hemisphere in the temporal lobe, the precuneus, and the paracentral lobule; and significant inverse relations are seen bilaterally in the cingulate and orbitofrontal cortex.

The ICV ∗ group interaction term was non-significant in all regions, thus this inverse relation between ICV and *nodal global efficiency* does not differ between individuals with ASD and typical controls.

**Figure 5:**
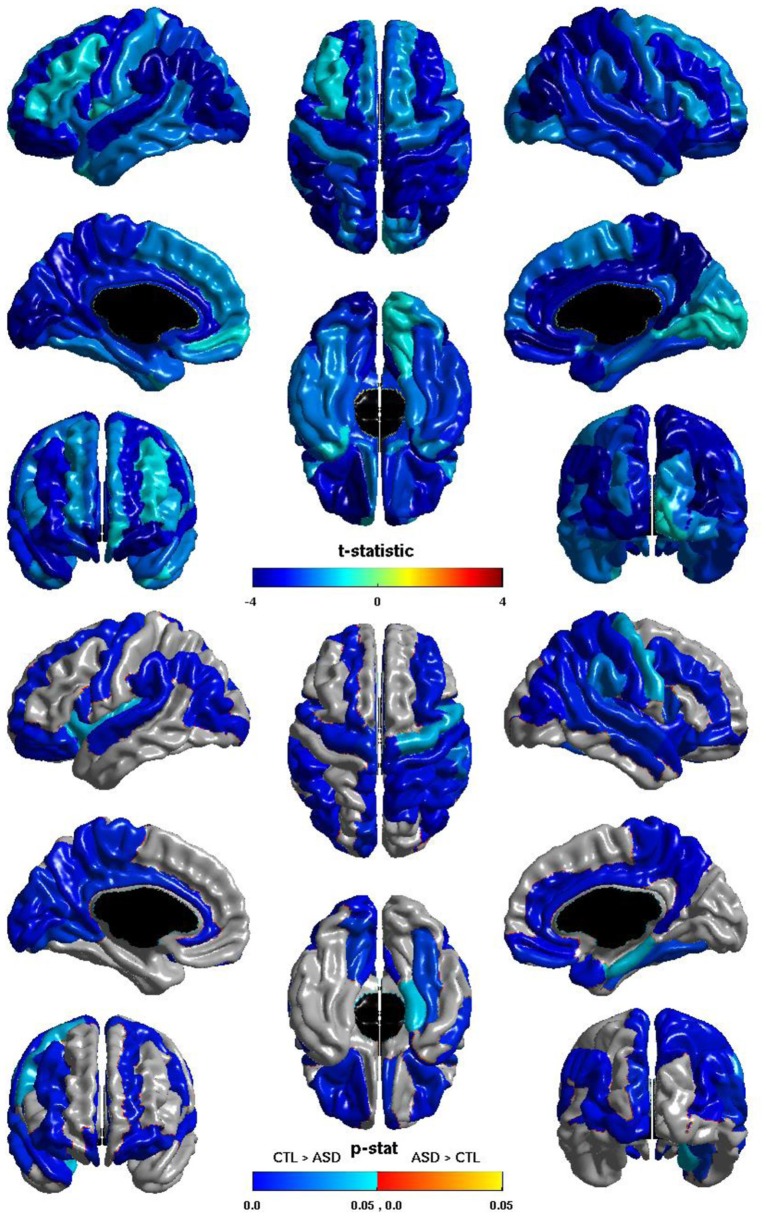
**Nodal Global Efficiency and Group.** The *t*-statistic **(top)** and the *p*-statistic **(bottom)** for the group difference in *nodal global efficiency* in each region of the AAL atlas. A negative *t*-statistic represents reduced efficiency in ASD. The *t*-statistic is negative everywhere. The *p*-statistic is FDR-corrected, and is blue where there is a significant reduction in *nodal global efficiency* in ASD, and orange where there is a significant increase in ASD. No regions show significantly increased *nodal global efficiency* in ASD. Significant reductions are seen bilaterally in all lobes. Note the similarities to the relation of *nodal global efficiency* and ICV.

The group differences in *nodal global efficiency* are shown in Figure 5. The *t*-statistic is negative over the entire cortex, thus for all regions *nodal global efficiency* is reduced in individuals with ASD. This reduction is significant over regions of all lobes in both hemispheres. Note that these reductions overlap with those of the relation of ICV and *nodal global efficiency* but are more extensive, particularly in the right hemisphere. The cosine similarity of the two *t*-statistic vectors is 0.9584.

Thus, both *nodal local efficiency* and *nodal global efficiency* showed an inverse relation to ICV, and in neither case was the ICV ∗ group interaction significant. Moreover, for both measures, the pattern of results for the inverse relation between ICV and efficiency was similar to the pattern of reductions in efficiency in ASD.

## Discussion

Networks with a high degree of spatially local connectivity, but with few long-range connections, i.e., shortcuts, have high local efficiency and low global efficiency; networks with a high degree of long-range connectivity, but which lack spatially local clustering, have high global efficiency and low local efficiency. Biological systems in general, and neural networks in particular, tend to balance global efficiency with local efficiency, having strong local clustering mixed with sufficient long-range connectivity to allow rapid communication between distant nodes; these have been dubbed “small-world” properties (Watts and Strogatz, 1998; Latora and Marchiori, 2001, 2003). The inverse relation shown here between ICV and both *nodal local* and *nodal global efficiency* suggests that deviation in brain growth trajectories impacts both long-range communication and within-neighborhood communication, and impacts both similarly. The absence of a group ∗ ICV interaction in either case indicates that the same is true in both typical adults and adults with ASD. The reductions in both *nodal local* and *nodal global efficiency* seen in individuals with ASD align with this inverse relation, in combination with the brain overgrowth that occurs in ASD, to suggest that the brain overgrowth may explain at least part of the reductions in efficiency; and the similarity of the spatial pattern of reductions in efficiency with the pattern of the relations between ICV and efficiency further supports this conclusion.

These results complement our previous work showing an inverse relation between the ICV-adjusted length of callosal fibers and degree of inter-hemispheric connectivity in ASD (Lewis et al., 2012), and our computational modeling work showing that the early brain overgrowth in ASD may cause the later reductions in long-range connectivity (Lewis and Elman, 2008). Those studies suggested that the brain overgrowth that occurs in ASD may underlie the reductions in long-range connectivity seen in adolescents and adults with ASD (Horwitz et al., 1988; Just et al., 2004, 2007; Kana et al., 2007). The current study extends that work to network analysis, relating the brain overgrowth in ASD to overall network organization.

The measures of efficiency utilized here do not directly correspond to connectivity; efficiency is defined in terms of paths through a network, not the strengths of individual connections. The network measures capture more complex aspects of brain organization. The inefficiencies in ASD shown here suggest a more random network organization, providing less well-segregated local processing and a reduced capacity to integrate information across the network. Reductions in *nodal global efficiency* might stem from either generally weaker connections, longer paths between nodes, or both. Topological measures show shorter characteristic path length in ASD (Rudie et al., 2013), meaning that communication between pairs of nodes is more direct. Together with the reductions in *nodal global efficiency* shown here this implies a more random configuration, with more but weaker shortcuts. The reductions in *nodal local efficiency* support this interpretation. Since the degree to which a node is a neighbor of another is determined by the strength of the direct connection between them, the neighbors of a node may be spatially distant. The local efficiency of a node thus reflects the spatial clustering of its neighbors, as well as the strength of the connections between them. Topological measures show reductions in modularity in ASD (Rudie et al., 2013), thus the reductions in *nodal local efficiency* in ASD should not be interpreted as short-distance under-connectivity, but as indicative of a more random configuration with more diffuse processing clusters. The inefficiencies in ASD thus suggest both less segregation and less integration. The inverse relation between the measures of efficiency and ICV suggests that these aspects of network organization are impacted by differences in brain growth trajectories.

This study complements the substantial body of research showing strong relationships between brain size and brain structure (Tower, 1954; Jerison, 1982; Ringo, 1991; Prothero, 1997; Zhang and Sejnowski, 2000; Karbowski, 2003; Changizi, 2007; Lewis et al., 2009). That research leaves unanswered the question of the aetiology of these scaling relationships. We have hypothesized that at least some of these scaling relationships come about over development as a consequence of the impact on normal developmental mechanisms of differences in metabolic costs and conduction delays associated with differences in brain size (Lewis and Elman, 2008; Lewis et al., 2012). Our hypothesis applies both to individual variability in growth trajectories in typical development, including gender differences, and to the atypical variations that are generally present in developmental disorders. The current results lend support to this conjecture.

ICV, however, is a very crude index of a very complex phenomenon. In typically developing infants the brain increases from approximately 25 percent of adult size at birth to approximately 75 percent of adult size by 2 years of age with substantial individual variability in rate of growth as well as mature brain size (Blinkov and Glezer, 1968; Dobbing and Sands, 1973; Courchesne et al., 2000). Multiple parameters are required to capture even the most basic aspects of such trajectories. ICV provides only an index of maximum brain size during development. Likewise, true efficiency of communication is determined by conduction delays and metabolic costs, and the measures used here serve as only crude proxies for such properties. The biophysical properties that determine conduction delays and metabolic costs, such as the density of fibers, axon diameters, and the degree of myelination, are only weakly related to the probabilistic tractography results used here as connection strengths. Further, the extent to which the results reported here are robust to the variety of factors that influence tractography-based estimates of connectivity, e.g., scan protocols, tractography parameters, and target parcellation (Jones et al., 2012), remains to be explored. The inverse relations between ICV and efficiency thus suggest that brain growth trajectories may account for a substantial part of the individual differences in brain organization both in typical adults as well as those with ASD, but the conjecture must be further tested utilizing methods which can provide more accurate estimates of brain growth trajectories, metabolic costs, and conduction delays.

### Conflict of interest statement

The authors declare that the research was conducted in the absence of any commercial or financial relationships that could be construed as a potential conflict of interest.
